# An Important Business Change

**Published:** 1883-10

**Authors:** 


### An Important Business Change.

Davidson's drug store, so long and favorably known to the profession of this city, has changed hands, having been purchased by Messrs. Shaver & Co. Dr. Davidson's skill as an analytical chemist is so well known throughout this and adjacent counties that his chemical work, together with the treatment of diseases of the kidneys and genito-urinary organs and diseases of the skin, to which he particularly devotes himself, have long made it impossible for him to give his personal attention to the drug business. In these important specialties of medicine, our esteemed editorial associate has had rare opportunities to perfect himself by his study in Philadelphia and London, and in them his chemical and microscopical knowledge are of peculiar service to him. In the department he has selected there is a wide and increasing field of work for the profession and of usefulness to the public, and we join in congratulations that he is now enabled to devote himself entirely to professional duties, which, though sufficiently arduous and exacting, are more congenial to his tastes, and in which he has already achieved merited success. The business assumed by

Messrs. Shaver & Co. comprises an old and well-established trade, which has always been noted for the purity and reliability of drugs dispensed. The public know so well the accomplished senior partner of the new firm, Mr. Shaver, who has won the confidence of the profession by his excellent pharmaceutical attainments, that an endorsement here is almost superfluous. We wish to commend him to all as especially deserving of patronage, which he will be sure to merit. The change, therefore, is in every respect advantageous to our confrere, and to the drug interests of our city. L.

